# Using near‐infrared reflectance spectroscopy (NIRS) to estimate carbon and nitrogen stable isotope composition in animal tissues

**DOI:** 10.1002/ece3.7851

**Published:** 2021-07-05

**Authors:** Francisco Javier Ancin‐Murguzur, Arnaud Tarroux, Kari Anne Bråthen, Paco Bustamante, Sébastien Descamps

**Affiliations:** ^1^ Department of Arctic and Marine Biology University of Tromsø – The Arctic University of Norway Tromsø Norway; ^2^ Norwegian Institute for Nature Research Tromsø Norway; ^3^ Littoral Environnement et Sociétés (LIENSs) UMR 7266 CNRS‐La Rochelle Université La Rochelle France; ^4^ Institut Universitaire de France (IUF) Paris France; ^5^ Norwegian Polar Institute Tromsø Norway

**Keywords:** dietary studies, isotopic analysis, NIRS

## Abstract

1. Stable isotopes analysis (SIA) of carbon and nitrogen provides valuable information about trophic interactions and animal feeding habits.
2. We used near‐infrared reflectance spectroscopy (NIRS) and support vector machines (SVM) to develop a model for screening isotopic ratios of carbon and nitrogen (*δ* ^13^C and *δ* ^15^N) in samples from living animals. We applied this method on dried blood samples from birds previously analyzed for *δ* ^13^C and *δ* ^15^N to test whether NIRS can be applied to accurately estimate isotopic ratios.
3. Our results show a prediction accuracy of NIRS (*R* ^2^ > 0.65, RMSEP < 0.28) for both *δ* ^13^C and *δ* ^15^N, representing a 12% of the measurement range in this study.
4. Our study suggests that NIRS can provide a time‐ and cost‐efficient method to evaluate stable isotope ratios of carbon and nitrogen when substantial differences in *δ* ^13^C or *δ* ^15^N are expected, such as when discriminating among different trophic levels in diet.

## Graphical abstract

In this article, we study how near infrared reflectance spectroscopy (NIRS) can be used to measure stable isotopes in animal tissues. We used seabird blood in our study and obtained satisfactory results for screening populations and discuss further use of NIRS in other tissues.

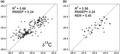

## 1 INTRODUCTION

The analysis of stable isotope ratios of carbon (*δ* ^13^C) and nitrogen (*δ* ^15^N) is a widely used approach in trophic studies at various ecological scales, from individuals to entire food webs (Boecklen, 2011; Navarro et al., 2011; Newsome et al., 2007). Stable isotope ratios in consumers’ tissues reflect those of their prey and of the relative proportion of each prey in the consumers’ diet (DeNiro & Epstein, 1978, 1981; Kelly, 2000). Through a single sampling event, the measurement of isotopic ratios can provide dietary information integrated over a period of time. The time period represented depends directly on the turnover rate of the tissue sampled and the species. It typically ranges from a few days in blood plasma, to several weeks in red blood cells, and up to several years in metabolically inert tissues such as bone collagen, teeth, or baleen (Hanson et al., 2009; Trueman et al., 2019).

Although isotopic analyses of animal tissues shed light onto the dietary habits of individuals and populations, they require the use of a mass spectrometer in specialized laboratories. As an alternative, near‐infrared reflectance spectroscopy (NIRS) can be used as a cost‐ and time‐effective method to measure tissue properties in animals and plants (Chodak, 2008; Cozzolino et al., 2001; Windley et al., 2013). NIRS is a nondestructive method where a beam of light with known properties is shed on the sample, which will absorb or reflect different wavelengths with varying intensity depending on the composition of the organic bonds (e.g., C–H, N–H, O–H) (Kaye, 1954, 1955). The reflectance or transflectance values are registered in the visible and near‐infrared spectrum (350–3000 nm), with the exact range and resolution dependent on the specific instrument used to measure the reflectance. The spectra obtained from the sample can subsequently be used to model other elements or molecules, given that a predictive model has been previously developed (Foley et al., 1998). The nondestructive nature of NIRS allows storing and reusing samples in further analyses, such as pollutant analyses. Although using NIRS as a method to estimate isotopic ratios is not new (Fuentes et al., 2012; Kleinebecker et al., 2009; Sun et al., 2012), to the best of our knowledge no study has explored the possibilities that NIRS offers for studying isotopic composition in wildlife animal populations.

In our study, we used blood samples previously collected in a study on a pelagic seabird, the Antarctic petrel (*Thalassoica antarctica*), to assess the suitability of NIRS to estimate stable isotopes in a free‐living animal species. Seabird ecology often relies on stable isotope analyses to estimate seabird diet or feeding habitat (Bond et al., 2016; Hovinen et al., 2019); a time and cost‐efficient method for the estimation of *δ* ^13^C and *δ* ^15^N would facilitate ecological research on seabirds and on more wildlife in general.

## 2 METHODS

### 2.1 Sampling and isotope measurements

Blood samples were collected during a previous study on Antarctic petrels (*Thalassoica antarctica*) at the Svarthamaren colony, Dronning Maud Land, Antarctica, and processed as described in Tarroux et al. (2020). Here, we used only the freeze‐dried red blood cells (hereafter RBC), but otherwise the analytical procedure in the laboratory is identical to that described in Tarroux et al. (2020). We evaluated the overall precision of measurement by duplicating a random subset of 10 RBC samples (Jardine & Cunjak, 2005). The mean absolute difference between duplicates was 0.05‰ for both *δ* ^13^C and *δ* ^15^N (range = [0; 0.11] and [0; 0.09], respectively).

### 2.2 NIRS scans

The freeze‐dried samples were poured into 4‐mm diameter custom‐made black polyacetal sample holders. The powder was subsequently pressed with a metal rod against the bottom glass to create an even surface and reduce light scattering. Scans were taken using a FieldSpec 3 (ASD Inc, Boulder, CO, USA) spectrometer, which has a spectral range of 350–2,500 nm. Spectra were registered at 1 nm interpolated resolution and saved as reflectance (R), with white reference measurements taken every 15 min to adjust for potential baseline drift. Each sample was scanned three times, rotating the sample holder between scans, to further reduce potential random light scattering effects.

### 2.3 Model development

All analyses were performed in R 3.5.1 (R Core Team, 2017). Model calibration was performed using support vector machines (SVM) contained in the *e1071* package (Meyer et al., 2015). Spectral treatments such as smoothing and derivatives, together with the standard normal variate (SNV) treatment, were applied to the spectral library to optimize the model development contained in the *prospectr* package (Stevens & Ramirez–Lopez, 2014). We developed the NIRS models excluding the visible light region (i.e., 350–720 nm) and the regions where the spectrometer sensors overlap (i.e., 950–1,050 nm, 1780–1,880 nm, and 2,450–2,500 nm).

The blood scan database (*n* = 213) was split into a calibration and validation dataset using the Kennard–Stone algorithm (Kennard & Stone, 1969) to ensure a high spectral variability included into the calibration dataset. We assigned 80% of the samples to the calibration dataset and 20% to the validation dataset, resulting in a calibration set of 171 samples and a validation set of 42 samples for both *δ* ^13^C and *δ* ^15^N.

We developed one distinct model for each isotope. Models were fitted using the calibration set and selected the kernel (i.e., linear, radial, polynomial, or sigmoid) based on its performance.

The SVM models were tested against the validation dataset: In addition to the *R* ^2^ value, we assessed the root mean squared error of the calibration (RMSEC) and the prediction (RMSEP), which measures the prediction error as integrated along the range of measured values with the formula:

$$\text{RMSE}=\sqrt{\sum_{i=1}^{n}\frac{{\left(\text{Predicted}‐\text{Measured}\right)}^{2}}{n}}$$

Furthermore, we assessed the range error ratio (RER), which is the ratio between the range for each calibration and the error (RMSEP). Models with RER values under 3 are considered unsuccessful, while RER values between 3 and 10 indicate limited applicability (e.g., screening) and RER values higher than 10 are considered to characterize high‐quality models (Quentin et al., 2017).

In addition, we computed the intercept and slope of the linear fit between predicted and measured values, as well as the bias (i.e., the systematic error of the predicted values) to estimate the suitability of NIRS to model *δ* ^13^C and *δ* ^15^N in future bird RBC samples.

## 3 RESULTS

**FIGURE 1:**
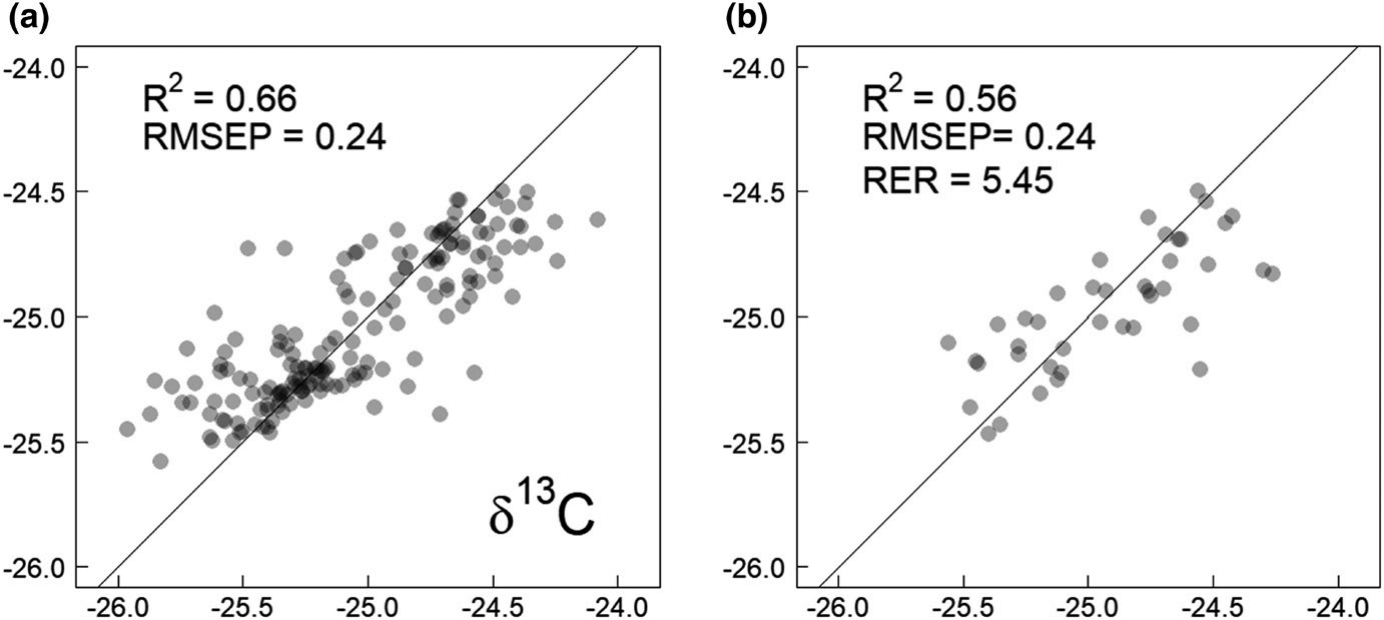
Calibration (a) and validation (b) plots for the *δ*13C model based on NIRS scans. The solid line indicates the perfect (1:1) fit

**FIGURE 2:**
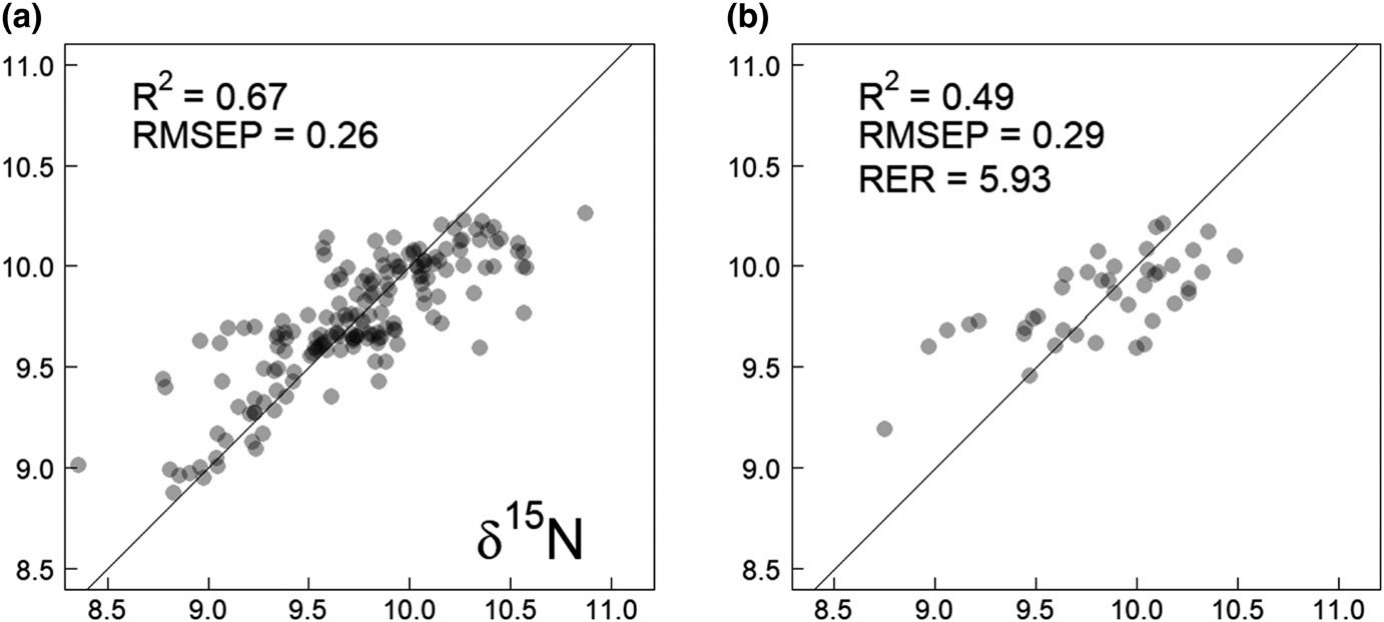
Calibration (a) and validation (b) plots for the *δ*15N model based on NIRS scans. The solid line indicates the perfect (1:1) fit

The RBC sample isotopic ratios ranged from −25.9 to −24.1 and from 8.4 to 10.9, respectively, for *δ* ^13^C and *δ* ^15^N: both the *δ* ^13^C ($R_{\text{validation}}^{2}$ = 0.56, RMSEP = 0.24, RER = 5.45) and the *δ* ^15^N ($R_{\text{validation}}^{2}$ = 0.49, RMSEP = 0.29, RER = 5.93) models showed similar performances. The RMSEP in both cases represented an error of approximately 12% of the total range (ranges are 1.88‰ for *δ* ^13^C and 2.51‰ for *δ* ^15^N), showing a linear relation between the measured and predicted values (Table 1, Figures 1 and 2).

**TABLE 1:** Prediction performance for *δ*13C and *δ*15N in the calibration and validation datasets using freeze‐dried bird RBC samples

Model	$R_{\text{cal}}^{2}$	RMSEC	$R_{\text{val}}^{2}$	RMSEP	Bias	Intercept	Slope	RER
*δ* ^13^C	0.66	0.24	0.56	0.24	0.05	1.78	1.07	5.45
*δ* ^15^N	0.67	0.26	0.49	0.29	−0.02	−3.37	1.34	5.93

Note

Eighty percent of the original dataset was used as the calibration dataset, while the remaining 20% was used for validation. $R_{\text{cal}}^{2}=R^{2}$ for calibration set, root mean squared prediction for the calibration (RMSEC) and validation (RMSEP) dataset, $R_{\text{val}}^{2}=R^{2}$ for validation set.

## 4 DISCUSSION

Our results show that NIRS can be a suitable tool to estimate stable isotopes in RBC samples from a free‐living animal population. Both *δ* ^13^C and *δ* ^15^N show an acceptable fit with RMSE values close to 0.25 and RER values in the acceptable range (5.45 for *δ* ^13^C and 5.93 for *δ* ^15^N), showing that NIRS can be used to estimate stable isotope compositions in animal tissues, although the model accuracy is not high enough to substitute laboratory‐grade measurements in studies addressing fine‐scale variation in isotopic ratios. It is important to note that the model performance is likely underestimated in our study, owing to the narrow range of values for isotopic ratios in our study species. To our knowledge, the only feasibility study of NIRS for isotopic estimation on animal tissues was performed on cattle meat: Sun et al. (2012) created predictive models for *δ* ^13^C and *δ* ^15^N, with a broader range of values in both elements compared to our study. Although their models had higher *R* ^2^ values than ours (again, likely due to the limited range of predictor values in our case), their RMSEP values were more than twice as large, suggesting that our models do in fact perform better. This difference can stem from the structural complexity of the tissue they used: muscle (meat) contains different cell types such as myocytes or adipocytes as well as connective tissue (e.g., collagen) and has a heterogeneous physical structure, while RBCs constitute a more homogeneous matrix in comparison (>99% erythrocytes). On the other hand, the relationship between C and *δ* ^13^C, and N and *δ* ^15^N, have been hypothesized to be an underlying reason for the good results in NIRS models for isotopes both in plant tissues (Hellmann et al., 2015) and soils (Winowiecki et al., 2017), although it has not yet been demonstrated. In our case, we found a nearly nonexistent correlation between *δ* ^13^C and C (*R* ^2^ = 0.04) and *δ* ^15^N and N (*R* ^2^ = 0.05) correlation in bird blood.

Several aspects make NIRS a very good candidate to estimate isotopic ratios of *δ* ^13^C and *δ* ^15^N in animal tissues. It is an easy‐to‐learn, quickly applicable tool: A short hands‐on training is enough to learn the basic use of the instrument (i.e., the scanning process), and the conversion from raw scans to isotopic values can be streamlined with a simple R‐code. Furthermore, since there is no need for reagents during the sample preparation or during the scanning process (other than the freeze‐drying), the cost of the analyses is reduced to acquiring the instrument and the initial investment for the model creation; the maintenance costs of the instrument are low. Afterward, only a subset of samples should be analyzed with the traditional method to ensure that the model continues to perform within the expected accuracy. This approach would allow to process much larger numbers of samples to detect outliers, on which further, more detailed, analyses could be conducted. An added advantage of NIRS is that the scanning and predicting process is very quick (up to 30 samples/hr). Once the reflectance readings are stored, there is no sample degradation, which allows for future chemical and isotopic analyses. Furthermore, a single NIRS scan can be used to estimate more than a single element or molecule (Ancin‐Murguzur et al., 2019; Foley et al., 1998). In this study, we developed models for *δ* ^13^C and *δ* ^15^N, but other models could be developed and used to predict other elements or even more complex molecules (Domján et al., 1994).

Although NIRS can be a cost‐ and time‐effective tool, its accuracy is lower than the reference method when it comes to SIA. Nevertheless, isotopic variability can be high among populations (Raya Rey et al., 2012) and also within populations (Martínez Del Rio et al., 2009) In such cases, this variability could be adequately captured by NIRS measurements. However, NIRS might not be able to identify subtle differences in isotopic composition among individuals or species with very, for example, closely related diets (Young et al., 2010), which would thus require proper SIA. That being said, the ability to increase the sample size with NIRS allows to obtain population‐level information rather than relying on lower sample size due to the economical and logistical constraints inherent in SIA. An alternative to using freeze‐dried blood is to use unprocessed blood, fresh blood: biomedical sciences routinely use NIRS for noninvasive blood analyses in screenings as a rapid analysis tool (e.g., Beć et al., 2020). The ability to measure blood properties without sample preparation could simplify the data gathering process to simply extracting blood drops from an individual and directly scan it, either in situ with a portable instrument or in the laboratory afterward. It remains to be tested if NIRS could be used for rapid, screening of population characteristics using other tissues such as feathers and hair. Such tissues could be scanned directly and noninvasively in the field (e.g., in the case of feathers of colonial seabirds, directly on the nest). This would substantially decrease the potential impact on animal welfare, while increasing the sample size.

## 5 CONCLUSION

Our study shows that NIRS is a time‐ and cost‐effective tool to estimate isotopic ratios of carbon and nitrogen in RBC samples, and we propose that it could be expanded to other tissues (e.g., feathers), leading to less invasive ecological studies on animals. Coupled with its portability and ease of use, NIRS can thus result in a highly useful and practical tool to better understand the trophic interactions between animals.

## CONFLICT OF INTEREST

The authors declare no conflict of interest.

## AUTHOR CONTRIBUTIONS

**Francisco Javier Ancin‐Murguzur:** Conceptualization (equal); Data curation (equal); Formal analysis (equal); Methodology (equal); Resources (equal); Software (equal); Validation (equal); Visualization (equal); Writing‐original draft (equal); Writing‐review & editing (equal). **Arnaud Tarroux:** Conceptualization (equal); Data curation (equal); Investigation (equal); Methodology (equal); Resources (equal); Supervision (equal); Validation (equal); Visualization (equal); Writing‐review & editing (equal). **Kari Anne Brathen:** Conceptualization (equal); Methodology (equal); Project administration (equal); Resources (equal); Writing‐review & editing (equal). **Paco Bustamante:** Formal analysis (equal); Methodology (equal); Resources (equal); Writing‐review & editing (equal). **Sébastien Descamps:** Data curation (equal); Funding acquisition (equal); Project administration (equal); Resources (equal); Supervision (equal); Validation (equal); Writing‐original draft (equal); Writing‐review & editing (equal).

## Data Availability

The spectra, reference values, and scripts are available at UiT open data repository: https://doi.org/10.18710/5PX1GJ.
